# Evaluation of rosuvastatin-induced QT prolongation risk using real-world data, in vitro cardiomyocyte studies, and mortality assessment

**DOI:** 10.1038/s41598-023-35146-z

**Published:** 2023-05-19

**Authors:** Yeryung Koo, Sung-Ae Hyun, Byung Jin Choi, Yujeong Kim, Tae Young Kim, Hong-Seok Lim, Joung-Wook Seo, Dukyong Yoon

**Affiliations:** 1 Department of Biomedical Informatics, Ajou University School of Medicine, Suwon, Gyeonggi-do Republic of Korea; 2 BUD.on Inc, Jeonju, Jeollabuk-do Republic of Korea; 3 Department of Advanced Toxicology Research, Korea Institute of Toxicology, KRICT, Daejeon, Republic of Korea; 4 Department of Biomedical Systems Informatics, Yonsei University College of Medicine, Yongin, Gyeonggi-do Republic of Korea; 5 Department of Cardiology, Ajou University School of Medicine, Suwon, Gyeonggi-do Republic of Korea; 6 Center for Digital Health, Yongin Severance Hospital, Yonsei University Health System, Yongin, Gyeonggi-do Republic of Korea; 7 Institute for Innovation in Digital Healthcare, Yonsei University, Seoul, South Korea

**Keywords:** Cardiovascular biology, Clinical pharmacology

## Abstract

Drug-induced QT prolongation is attributed to several mechanisms, including hERG channel blockage. However, the risks, mechanisms, and the effects of rosuvastatin-induced QT prolongation remain unclear. Therefore, this study assessed the risk of rosuvastatin-induced QT prolongation using (1) real-world data with two different settings, namely case–control and retrospective cohort study designs; (2) laboratory experiments using human-induced pluripotent stem cell-derived cardiomyocytes (hiPSC-CM); (3) nationwide claim data for mortality risk evaluation. Real-world data showed an association between QT prolongation and the use of rosuvastatin (OR [95% CI], 1.30 [1.21–1.39]) but not for atorvastatin (OR [95% CI], 0.98 [0.89–1.07]). Rosuvastatin also affected the sodium and calcium channel activities of cardiomyocytes in vitro. However, rosuvastatin exposure was not associated with a high risk of all-cause mortality (HR [95% CI], 0.95 [0.89–1.01]). Overall, these results suggest that rosuvastatin use increased the risk of QT prolongation in real-world settings, significantly affecting the action potential of hiPSC-CMs in laboratory settings. Long-term rosuvastatin treatment was not associated with mortality. In conclusion, while our study links rosuvastatin use to potential QT prolongation and possible influence on the action potential of hiPSC-CMs, long-term use does not show increased mortality, necessitating further research for conclusive real-world applications.

## Introduction

Drug treatment can prolong QT interval by blocking the cardiac potassium channel, also known as the human Ether-à-go-go-Related Gene (hERG) channel^1^, thereby reducing potassium ion influx and prolonging ventricle repolarization^2^. Since 2005, several international regulatory agencies have recommended testing the inhibitory effect of new drugs on the hERG channel in preclinical settings. However, recent studies have revealed various mechanisms underlying QT prolongation besides blocking the hERG channel^1,3^. The multiple ion channel effect model is superior to the hERG channel assay, in which sodium and calcium channel blocking is also assayed to assess the risk of QT prolongation^4^. However, many currently available drugs are only assessed using the hERG assay at the preclinical level. Hence, studies have attempted to assess the risk of drug-induced QT prolongation at the clinical level. For example, although CredibleMeds.org has made an effort to provide the most recent list of QT drugs^5^, drugs with unknown risks of QT prolongation remain commonly prescribed.

We have previously used a data-driven method to retrospectively analyze 167 drugs with unknown risks of QT prolongation frequently used in a tertiary teaching hospital in South Korea^6^ and identified 38 drugs with a possible risk of QT prolongation. These results provided evidence regarding the risk of using azithromycin, a candidate drug to treat patients with COVID-19^7,8^.

Our previous findings also showed contrasting results between rosuvastatin and atorvastatin, the most common statins used for treating hyperlipidemia^9,10^; in clinical settings, rosuvastatin is associated with an increased risk of QT prolongation, whereas atorvastatin is not. Although rosuvastatin has been approved in 156 countries, there remain conflicting results regarding its safety. Some studies have shown that rosuvastatin blocks the hERG channel and prolongs QT intervals in vitro, ex vivo, and in vivo^11,12^. Meanwhile, studies examining the blockade of calcium and sodium channels by rosuvastatin are currently lacking.

Therefore, in this study, we comprehensively assess the risk of rosuvastatin-induced QT prolongation. First, we assessed the risk of rosuvastatin-induced QT prolongation using a real-world setting by retrospectively analyzing data from a medical database spanning 22 years containing electrocardiogram (ECG) data from a tertiary teaching hospital. Second, we examined the action potential in human-induced pluripotent stem cell-derived cardiomyocytes (hiPSC-CMs) to assess the inhibitory effect of rosuvastatin on potassium, calcium, and sodium channels. Third, we used data spanning 15 years from the National Health Insurance Service (NHIS) database of South Korea to evaluate the effect of rosuvastatin or atorvastatin on the hard endpoint of all-cause mortality.

## Methods

**Figure 1:**
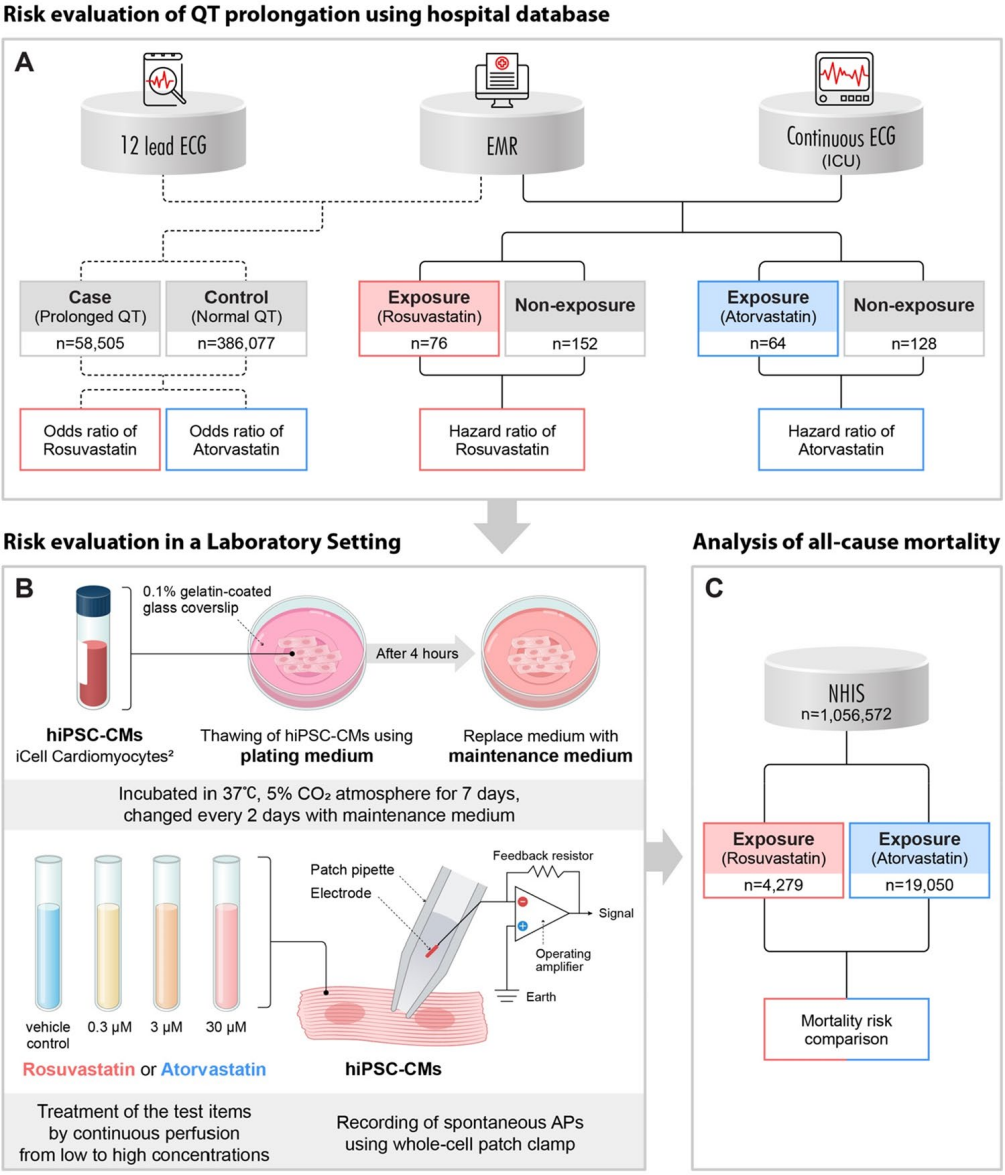
Overview of the study process. For QT prolongation risk evaluation of rosuvastatin and atorvastatin, real-world data were analyzed via two different study designs (case–control and retrospective cohort study). Laboratory experiments were conducted to confirm whether rosuvastatin or atorvastatin affect cardiac action potential in hiPSC-CMs. To evaluate the effect on mortality, nationwide claim data were analyzed. EMR, electronic medical record; ICU, intensive care unit; HiPSC-CM, human-induced pluripotent stem cell-derived cardiomyocytes; NHIS, National Health Insurance Service.

As illustrated in Fig. 1, this study consisted of three steps: (1) risk evaluation in real-world data; (2) risk evaluation in a laboratory setting; and (3) analysis of the NHIS database from South Korea. The Institutional Review Board of Ajou University Medical Center approved this study (protocol AJIRB-MED-MDB-19-406) and waived the requirement for informed consent since only anonymized data were used retrospectively. All methods were performed in accordance with the relevant guidelines and regulations.

### Risk evaluation from real-world data

#### Case–control design-based study

A case–control study was designed to assess the risk of rosuvastatin-induced QT prolongation compared with that of atorvastatin using a database from a tertiary teaching hospital in South Korea (Ajou University Hospital). This included the ECG database, which contains 1,040,752 ECG results of standard 10-s 12-lead ECGs from June 1994 to August 2018. The QTc values were extracted from the local ECG repository in the MUSE™ system^13^. QT prolongation was defined as QTc (calculated using the Bazett formula) > 450 ms for males and 460 ms for females^14,15^. Each QTc value was integrated with the electronic medical record (EMR) data using patient ID to obtain information regarding patient demographics, laboratory test results, comorbidities, and drug prescriptions. The EMR database contained 177,841,556 drug prescriptions, 379,994,144 laboratory test results, and 3,024,891 patient demographics from 1994 to 2018.

To select the case and control groups, one ECG result was randomly selected for each patient, and 444,632 ECG results were extracted (Fig. S1). The age and sex information of 3,050 patients were not available; therefore, we excluded these patients from the analysis; a total of 444,582 patients were included in the analysis, of which the case group with QT prolongation comprised 58,505 patients, while the control group comprised 386,077 patients.

To adjust for bias, the following confounding variables were included in the analysis: sex, age, serum potassium and calcium levels within a year prior to ECG measurement, comorbidities that are known risk factors for QT prolongation within one year prior to the ECG measurement extracted using the International Classification of Diseases (ICD-10) codes (Table S1), and frequency of usage of drugs with known risk of QT prolongation within 7 d prior to ECG measurement.

The list of drugs with known risk of QT prolongation was cited from CredibleMeds®^16,17^. The QT drug list categorizes drugs into four classes based on the strength of evidence regarding causality (risk): known risk of Torsades de pointes (TdP) (rank 1), possible risk of TdP (rank 2), conditional risk of TdP (rank 3), and use of medications to avoid congenital long QT syndrome (rank 4). The frequencies of drugs in each category were counted and used as confounding variables. The total frequency of drug use in the four categories was also calculated and used as a separate variable. The full list of drugs in all categories is listed in Table S2.

Using the variables described above, the adjusted odds ratio of exposure to rosuvastatin or atorvastatin in QT prolongation events was evaluated.

#### Retrospective cohort design-based study

The risk of QT prolongation in patients treated with rosuvastatin or atorvastatin was investigated and compared with that in unexposed patients. As continuously observed ECG data are required to perform cohort design-based studies, the EMR and continuous ECG monitoring data of patients admitted to the intensive care unit (ICU) between March 2017 and May 2018 were included.

While selecting the subject group among the patients administered rosuvastatin in the ICU between March 2017 and May 2018, patients with missing sex and age information (n = 331), missing ECG monitoring data (rosuvastatin n = 450, atorvastatin n = 254), experienced QT prolongation before using rosuvastatin (rosuvastatin n = 45, atorvastatin n = 47), and those without ECG monitoring within 6 h of the last drug use (rosuvastatin, n = 9; atorvastatin, n = 9) were excluded. QT prolongation was defined as QTc (calculated using the Bazett formula) > 450 ms for males and 460 ms for females, as defined in the case–control study design. For the unexposed group for each subject, the 1:2 ratio was matched with the variables of sex, age, and duration of ICU stay. In this matching process, four patients in the rosuvastatin group and 16 in the atorvastatin group were excluded. Finally, 76 exposed and 152 unexposed patients were included in the rosuvastatin analysis (Fig. S2), while 64 exposed and 128 unexposed patients were included in the atorvastatin analysis (Fig. S3).

The last drug use was defined as no additional drug use for 48 h, which was twice the average drug administration period in the subject hospital. The ECG monitoring data was tracked until 24 h after the last drug use, termination of ECG monitoring upon QT prolongation, or discontinuation of ECG monitoring for longer than 6 h.

To adjust for bias, the following confounding variables were included: (1) serum calcium and potassium levels within one year prior to ECG measurement, (2) comorbidities known as risk factors of QT prolongation, and (3) use of concomitant drugs known to prolong the QT interval (Tables S3 and S4)^18^. When the comorbidities for each patient were investigated, the ICD-10 codes within one year prior to the drug exposure date based on the EMR data were extracted. To define concomitant drug use, an average half-life of 6 h was applied to reflect the drug use between 6 h prior to the target drug administration (rosuvastatin or atorvastatin) and the end of follow-up. In this analysis, the adjusted hazard ratio (HR) of rosuvastatin or atorvastatin exposure was evaluated.

### Risk evaluation in a laboratory setting

#### Cardiomyocyte cell culture

The hiPSC-CMs (Cellular Dynamics International [CDI], Madison, WI, USA) were prepared according to the manufacturer’s instructions. For manual patch clamp recording, hiPSC-CMs were thawed and plated onto coverslips coated with 0.1% gelatin solution. After incubation at 37 °C and 5% CO_2_ for 4 h, the plating medium was replaced with maintenance medium, which was changed every 2 d. The hiPS-CMs were cultured for 14 d and used on days 7–14 for electrophysiological analysis.

#### Whole-cell voltage-clamp recordings

Whole-cell recordings of hiPSC-CMs were performed as previously described^19^. A coverslip was placed in a perfusion chamber at 37 °C. The extracellular solution contained the following: 15 mM HEPES, 5.4 mM KCl, 150 mM NaCl, 1 mM sodium pyruvate, 1 mM MgCl_2_, 1.8 mM CaCl_2_, and 15 mM glucose (pH 7.4). The internal solution contained the following components: 2 mM CaCl_2_, 5 mM EGTA, 10 mM HEPES, 150 mM KCl, 5 mM Mg-ATP, and 5 mM NaCl (pH 7.25). The test drugs were prepared as follows: To prepare 1000 × stock solution, the drug was dissolved with DMSO; for dose formulation, the 1000 × stock solution was diluted in extracellular solution to produce the exposure concentrations 0.3, 3, and 30 µM. The typical action potentials (APs) in hiPSC-CMs were recorded in the current-clamp mode and the test drug solution superfused for approximately 4 min in serial order from the lowest to the highest concentration. Following the stabilization of AP waveforms, the average of five recorded APs for each test concentration was analyzed. AP parameters, including maximum diastolic potential (MDP), action potential amplitude (APA), maximum upstroke velocity (dV/dt_max_), APD at 50% repolarization (APD_50_), and APD at 90% repolarization (APD_90_), were analyzed and quantified.

### Analysis of all-cause mortality

The national claims data were used to determine whether the use of atorvastatin or rosuvastatin was associated with death. The data were provided by NHIS, which manages the entire claims data in the South Korean population. The NHIS database includes more than 100 variables, such as age, sex, disease type, prescriptions, and health screening records. For this study, a customized database that included claims data from patients prescribed either rosuvastatin or atorvastatin between January 2005 and December 2019 was used. The total population was 2,121,500 before applying the exclusion criteria. Patients who received the same drug within six months following the initial prescription were selected; finally, 1,056,572 patients were selected after excluding 1,064,928 patients (Fig. S4). Mortality events during drug administration were assessed; if a patient's date of death was earlier than the last date of drug use, it was considered a mortality case possibly related to drug use.

During the year before the first drug use (rosuvastatin or atorvastatin), the Charlson and Elixhauser comorbidity index was calculated^20,21^. The number of drugs that can cause QT prolongation for each patient was determined using the information in “Crediblemeds.org.” Finally, the adjusted HR of exposure to rosuvastatin or atorvastatin was evaluated.

### Statistical analysis

The *t*-test was performed for continuous variables, while Pearson’s chi-square test was used for categorical variables to analyze the difference between baseline characteristics of the case *vs*. control groups and the exposed *vs*. unexposed groups. Fisher’s exact test was performed when the chi-square test was not applicable owing to a lack of counts.

Multiple logistic regression was performed to compare the risk of rosuvastatin- or atorvastatin-induced QT prolongation between the case and control groups. To compare the risk of QT interval prolongation between the statin-exposed and unexposed groups, the Cox proportional hazards model was used to calculate the adjusted HR using confounding variables. The Cox proportional hazards model was also used for mortality analysis.

Statistical significance was set at *p*-value < 0.05. Data management was performed using Azure Data Studio version 1.19.0 and SAS Enterprise Guide 7.1. Statistical analyses were conducted using the Python packages statsmodels version 0.11.1 and pymatch version 0.3.4, as well as R version 4.0.3.

For risk evaluation in the laboratory setting, data were analyzed using GraphPad InStat version 3.0 (GraphPad Software, San Diego, CA, USA) and presented as mean ± standard error of the mean. Data were analyzed using analysis of variance, while multiple comparisons among groups were assessed using Dunnett's test. Statistical significance was set at a *p*-value less than 0.01 or 0.001.

## Results

### Risk evaluation in real-world data

#### Case–control design-based study

Table S5 lists the baseline characteristics of the QT prolongation case and control groups. The total number of included patients was 444,582 (Fig. 1), of which 58,505 (13.15%) and 386,077 (86.84%) patients belonged to the QT prolongation and control groups, respectively. The proportion of patients using rosuvastatin within 7 d prior to the ECG measurement date in the QT prolongation group was higher than that in the control group (2.7% *vs*. 0.9%). The proportion of subjects using atorvastatin in the QT prolongation group was higher than that in the control group (1.3% *vs*. 0.6%) but lesser than that in the rosuvastatin group. The proportion of QT prolongation was higher in males, elderly patients, and patients with lower potassium and calcium levels.

Table 1 lists the results of the multiple logistic regression analysis, which assessed QT prolongation associated with the use of rosuvastatin and atorvastatin. Rosuvastatin use was more likely to be associated with QT prolongation (OR [95% CI], 1.30 [1.21–1.39]), whereas atorvastatin use was not associated with QT prolongation (OR [95% CI], 0.98 [0.89–1.07]).

**Table 1:** Results of multiple logistic regression analysis.

	OR	95% CI	*p*-Value
Rosuvastatin	1.30	1.21–1.39	< 0.001
Atorvastatin	0.98	0.89–1.07	0.5837
Male (ref. female)	1.09	1.07–1.11	< 0.001
Age (ref. under 39)			
40–49	1.45	1.40–1.49	< 0.001
50–59	1.95	1.89–2.01	< 0.001
60–69	2.50	2.43–2.57	< 0.001
70–79	3.62	3.51–3.74	< 0.001
80–89	4.89	4.68–5.10	< 0.001
≥ 90	7.41	6.56–8.37	< 0.001
Serum potassium level (ref. 0–3.4 mEq/L)			
3.5–3.7 mEq/L	0.61	0.58–0.64	< 0.001
3.8–4.0 mEq/L	0.43	0.41–0.45	< 0.001
4.1–4.3 mEq/L	0.34	0.33–0.36	< 0.001
4.4–4.6 mEq/L	0.32	0.31–0.34	< 0.001
4.7–4.9 mEq/L	0.33	0.31–0.35	< 0.001
≥ 5.0 mEq/L	0.31	0.30–0.33	< 0.001
Serum calcium level (ref. 0–8.4 mg/dL)			
8.5–8.8 mg/dL	0.65	0.63–0.67	< 0.001
8.9–9.2 mg/dL	0.55	0.53–0.56	< 0.001
9.3–9.6 mg/dL	0.54	0.52–0.56	< 0.001
9.7–10.0 mg/dL	0.57	0.55–0.60	< 0.001
10.1–10.4 mg/dL	0.61	0.59–0.64	< 0.001
≥ 10.5 mg/dL	0.68	0.61–0.76	< 0.001
Comorbidity			
Myocardial infarction	1.53	1.43–1.64	< 0.001
Congestive heart failure	2.16	2.02–2.32	< 0.001
Ischemic stroke	1.48	1.40–1.56	< 0.001
Hemorrhagic stroke	2.08	1.93–2.24	< 0.001
Diabetes mellitus	1.11	1.06–1.16	< 0.001
Hypothyroidism	0.92	0.80–1.06	0.2548
Renal disease	2.61	2.45–2.78	< 0.001
AIDS/HIV	1.36	0.85–2.18	0.2056
Alcohol abuse	2.62	2.36–2.91	< 0.001
Drug abuse	3.15	2.62–3.80	< 0.001
Liver disease	2.11	1.93–2.31	< 0.001
Severe liver disease	2.74	2.30–3.25	< 0.001
Drug use total by rank (ref. 0)			
Rank 1 drug	0.96	0.91–1.02	0.1940
Rank 2 drug	1.07	1.01–1.13	0.0331
Rank 3 drug	0.89	0.84–0.95	0.0004
Rank 4 drug	0.85	0.80–0.90	< 0.001
Drug use total (ref. 0)			
1	1.73	1.62–1.84	< 0.001
2	2.24	1.99–2.53	< 0.001
3	2.76	2.31–3.30	< 0.001
4	3.26	2.57–4.13	< 0.001
5	3.78	2.81–5.08	< 0.001
6	4.17	2.92–5.96	< 0.001
7	4.74	3.13–7.19	< 0.001
8	4.93	3.06–7.94	< 0.001
9	6.33	3.70–10.84	< 0.001
Over 10	6.93	3.55–13.53	< 0.001

#### Retrospective cohort design-based study

The total number of patients enrolled in the study for rosuvastatin and atorvastatin analysis was 228 and 192, respectively (Fig. 1). The characteristics of the subjects in the exposed and unexposed groups are listed in Tables S3 and S4.

Table 2 lists the results of the Cox regression analysis for rosuvastatin and atorvastatin. We removed the variables with a lack of counts in each analysis. The HR of rosuvastatin was > 1 (HR: 2.54, 95% CI: 0.94–6.82), whereas that of atorvastatin was < 1 (HR: 0.57, 95% CI: 0.19–1.75). Although these results were not statistically significant due to insufficient patient numbers, QT interval prolongation risk was associated with rosuvastatin use with marginal significance (*p* = 0.0647).

**Table 2:** Results of cox regression analysis for rosuvastatin and atorvastatin.

	Rosuvastatin-induced QT prolongation			Atorvastatin-induced QT prolongation		
	HR	95% CI	*p*-value	HR	95% CI	*p*-Value
Rosuvastatin	2.54	0.94–6.82	0.0647	–	–	–
Atorvastatin	–	–	–	0.57	0.19–1.75	0.3283
Serum potassium level (ref < 3.5 mEq/L)						
3.5–3.8 mEq/L	0.46	0.15–0.44	0.1181	3.94	0.64–23.23	0.1393
3.8–4.1 mEq/L	0.32	0.10–1.04	0.0573	2.16	0.39–11.94	0.3758
4.1–4.4 mEq/L	0.50	0.14–1.74	0.2738	3.61	0.67–19.38	0.1341
4.4–4.7 mEq/L	0.53	0.10–2.73	0.4477	3.66	0.50–26–69	0.2005
4.7–5.0 mEq/L	0.12	0.01–1.34	0.0858	4.78	0.70–32.50	0.1094
> 5.0 mEq/L	–	–	–	9.15	1.17–71.53	0.0348*
Serum calcium level (ref < 8.5 mg/dL)						
8.5–8.9 mg/dL	1.64	0.56–4.81	0.3644	1.41	0.45–4.40	0.5510
8.9–9.3 mg/dL	2.06	0.71–6.00	0.1856	1.30	0.38–4.38	0.6749
9.3–9.7 mg/dL	1.01	0.25–4.01	0.9920	1.05	0.26–4.22	0.9481
> 9.7 mg/dL	–	–	–	0.87	0.09–7.93	0.8988
Comorbidity						
Myocardial infarction	0.51	0.13–1.91	0.3162	3.05	0.60–15.33	0.1768
Congestive heart failure	1.15	0.26–5.15	0.8561	–	–	–
Ischemic stroke	2.68	0.39–18.38	0.3145	1.17	0.25–5.59	0.8429
Hemorrhagic stroke	–	–	–	3.93	0.98–15.78	0.0539
Diabetes mellitus	0.83	0.23–2.94	0.7730	0.78	0.16–3.80	0.7538
Renal disease	4.92	1.46–16.57	0.0102*	3.69	0.68–19.90	0.1292
Drug abuse	5.53	0.57–53.43	0.1397	-	-	-
Concomitant drugs						
Fluoroquinolone antibiotics	0.56	0.07–4.75	0.5920	–	–	–
Macrolide antibiotics	0.29	0.02–3.93	0.3491	–	–	–
Anesthetic/sedative	2.60	0.70–9.70	0.1555	1.20	0.28–5.09	0.8060
Gastrointestinal promotility	0.39	0.08–1.90	0.2455	–	–	–
Antipsychotics	0.24	0.03–2.08	0.1963	1.42	0.28–7.22	0.6737
Vasodilator drugs	0.38	0.02–6.27	0.4991	–	–	–
Antiarrhythmic	–	–	–	3.50	0.37–32.95	0.2742
SSRI^a^	–	–	–	5.49	0.75–40.17	0.0937

Significance codes: 0 “***” 0.001 “**” 0.01 “*” 0.05 “.” 0.1 “ ” 1.

^a^Selective serotonin reuptake inhibitors.

### Risk evaluation in a laboratory setting

**Figure 2:**
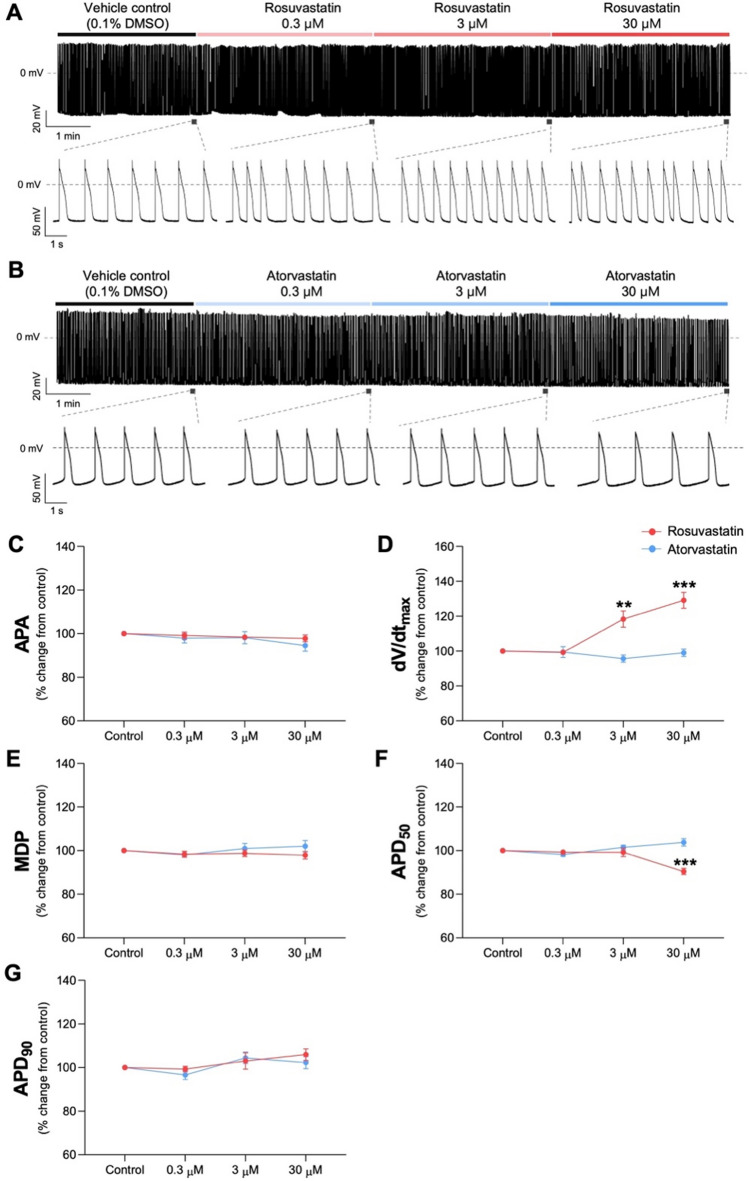
Effects of rosuvastatin and atorvastatin on action potential waveforms. Representative traces of rosuvastatin and atorvastatin on action potential (AP) are shown (**A** and **B**). The normalized AP parameters were quantified following treatment with 0.3, 3, and 30 µM rosuvastatin or atorvastatin (**C**–**G**). APA, action potential amplitude; dV/dt_max_, maximum upstroke velocity; MDP, maximum diastolic potential; APD_50_, action potential duration at 50% repolarization; APD_90_, action potential duration at 90% repolarization; n = 5 independent cultures for each condition. Statistical significance was determined using one-way analysis of variance with Dunnett’s test. Data are expressed as percentage inhibition concentration and are shown as mean ± standard error of the mean. **p < 0.01 and ***p < 0.001 compared to the control.

Figure 2 shows the response of hiPSC-CM APs to rosuvastatin or atorvastatin (Fig. 2). AP parameters obtained upon treatment with either rosuvastatin or atorvastatin were normalized to the control values in each cell. Rosuvastatin treatment did not affect the APA, MDP, and action potential duration at 90% repolarization (APD_90_) but increased the maximum upstroke velocity (dV/dt_max_) from 3 µM (Fig. 2 D) and decreased the APD_50_, action potential duration at APD_50_ from 30 µM (Fig. 2F). However, atorvastatin did not significantly affect AP parameters up to 30 µM. These results indicate that rosuvastatin has a more potent effect on cardiac action potential compared with atorvastatin.

### Analysis of the data from the NHIS database

Table S6 lists the baseline characteristics of patients who received rosuvastatin or atorvastatin based on NHIS. Among 1,056,572 patients, 28% received rosuvastatin, and 2.21% died following exposure to either drug. The number of patients who died after receiving rosuvastatin was 4,279 (1.4%), and the number of patients who died after receiving atorvastatin was 19,050 (2.5%). Liver and cerebrovascular diseases were the most common comorbidities affecting 416,048 and 162,014 patients, respectively.

Table S7 lists the results of the Cox proportional hazards model. Rosuvastatin treatment did not show significant HR with *p* > 0.05 (HR [95% CI], 0.95 [0.89–1.01]). However, the greater the number of drugs from the CredibleMeds drug list that a patient receives, the more likely they are to experience QT prolongation (HR [95% CI], 1.01 [1.01–1.01], *p* < 0.001).

## Discussion

In this study, we observed a higher risk of QT prolongation in real-world data associated with treatment with rosuvastatin than atorvastatin. The difference was significant in the case–control study design (all inpatients and outpatients) and retrospective cohort study design (including patients in the ICU). Furthermore, rosuvastatin affected the action potential of hiPSC-CMs in laboratory settings and hindered the sodium and calcium channels rather than the potassium channel. However, no significant association was observed between all-cause mortality and rosuvastatin use in a nationwide study.

As a commonly used statin, rosuvastatin, first approved by the Food and Drug Administration in 2003, is used to treat hyperlipidemia^22^. Rosuvastatin has since been approved in 156 countries. In 2019, rosuvastatin ranked 21st among the most commonly used drugs, according to the Drug Usage Statistics, United States of America (USA), 2013–2019. The estimated number of prescriptions for rosuvastatin in the USA is 27,041,319^23^. As its effectiveness and safety have been proven in numerous clinical studies, credible international guidelines are highly recommended for treating hyperlipidemia using rosuvastatin.

Rosuvastatin showed a significant association with the risk of QT prolongation (*p* < 0.05), whereas atorvastatin showed no such association^24,25^. However, rosuvastatin was not significantly associated with all-cause mortality in the risk assessment based on NHIS data, which may be explained by the cardioprotective effect of statins^26^, suggesting that the effect of rosuvastatin on the hard endpoint of mortality is not significant in the long-term, which is in agreement with previous findings^27–29^. Therefore, although an association with QT prolongation was confirmed in this study, it may not be a type of QT prolongation that leads to a further serious adverse reaction.

Furthermore, a previous randomized, double-blind, placebo-controlled trial was conducted at 52 centers in North America to compare the effects of rosuvastatin and atorvastatin. The authors reported that rosuvastatin was more effective in controlling lipoprotein cholesterol levels in patients with hypercholesterolemia than atorvastatin^9^. As the clinical benefits of rosuvastatin have been previously demonstrated, rosuvastatin should be used with active monitoring for QT prolongation in cases with an elevated risk of serious QT prolongation, such as comorbidities or concomitant medications, in which the use of other statins, such as atorvastatin, should be considered.

The possible risk of rosuvastatin-induced QT prolongation observed in real-world data implies that several drugs with unknown risk of QT prolongation are used in clinical settings. Since 2005, the safety of drugs at the preclinical stage has only been examined based on hERG channel inhibition. However, drugs that have passed the hERG test and been marketed have been withdrawn due to side effects, including cardiac toxicity^30^. Furthermore, utilization of hiPSC-CMs for the assessment of QT prolongation is important, as indicated by the comprehensive in vitro proarrhythmia assay paradigm that proposes to evaluate proarrhythmic risk based on a mechanistic electrophysiologic understanding of proarrhythmia^31^. Therefore, we evaluated the effect of rosuvastatin and atorvastatin on cardiac action potential using hiPSC-CMs cells. When we examined the action potential following rosuvastatin treatment, dV/dt_max,_ associated with activation of the sodium channel, showed a significant increase at 3 and 30 µM, while APD_50,_ associated with activation of the calcium channel, showed a significant reduction at 30 µM (Fig. 2 D and F). These results suggest that rosuvastatin affects calcium and sodium channels, further supporting the multiple ion channel effect model and highlighting the necessity of assaying multiple ion channels to assess the risk of QT prolongation at the preclinical stage. It is critical to assess the risk of QT prolongation for drugs commonly used in clinical trials since the risk assessment for many remains lacking. In our previous study, we identified that 37 drugs, other than rosuvastatin, showed potential for QT prolongation risk using a data-driven method^6^.

However, the pathophysiology underlying the experimental and clinical results requires further elucidation. Specifically, the experiment demonstrated an increased heart rate by affecting calcium channels, although this effect was not observed in other randomized controlled trials^32,33^. Establishing a direct link between laboratory and clinical results remains necessary. Nonetheless, our study provides evidence of a mild QT interval increase in the real world with rosuvastatin use and that rosuvastatin affected the calcium channel, one of several contributing factors to QT interval prolongation. Further research is needed to understand the pathophysiological principles underlying the observed results from both analyses.

Our results for other variables were consistent with previous findings. In the regression model, older age, and cardiovascular diseases, such as myocardial infarction, ischemic stroke, and hemorrhagic stroke, were associated with a higher risk of QT prolongation (Table 1). Diabetes mellitus, renal disease, liver disease, and the frequency of use of QT prolongation-associated medications were also associated with QT prolongation. The odds ratio of QT prolongation increases with the number of medications administered. In the all-cause mortality analysis, most comorbidities, except for AIDS, alcohol abuse, and moderate to severe liver disease, showed significant HRs, although exposure to rosuvastatin was not associated with significant HR (Table S7). The renal disease showed the highest HR with a significant *p*-value (HR [95% CI], 2.22 [2.04–2.43]; *p* < 0.001).

This study has some limitations. First, we used data from only one institution for the real-world data analysis, which limited the number of patients in the study; we found no significant HR for rosuvastatin in the retrospective cohort design study; however, the *p*-value was close to 0.05 (HR: 2.54, *p*-value: 0.0647). We observed a trend for increased risk of QT prolongation, demonstrating the necessity of further studies with larger cohort size. Moreover, we could not evaluate the incidence of TdP, one of the most serious outcomes led by QT prolongation and mortality in the subject hospital. The incidence of TdP is rare and not well-documented in electronic medical records. Because the subject hospital is a tertiary teaching hospital that mainly treats acute and severe stages of the disease, many patients die outside of the subject hospital, mainly after being transferred to the community hospital. To overcome this limitation, we tried to evaluate the effect on mortality using a national claim dataset in this study.

Second, we only focused on the occurrence of the event, whether the QT interval exceeded the specific cutoff or not. Due to the limitation of our dataset, in which the time interval between two consecutive ECG examinations was too extended in the case–control study^34^ and that we must select a representative value from the continuous waveform, we could not calculate the effect size of atorvastatin and rosuvastatin on QT prolongation. To clarify the clinical impact of QT prolongation driven by rosuvastatin, further studies to measure the effect size of the drug are warranted. However, according to previous reports that tried to measure the effect size of rosuvastatin with guinea pigs, the effect size on QTc prolongation of rosuvastatin was about 4–5% (from 201 ± 1 to 210 ± 2 ms, *p* < 0.05)^11^. Another limitation of our study is that we could not evaluate the exposure of our target drugs and cardiovascular death rather than all-cause mortality, as our dataset did not provide information about the cause of death. Moreover, we could not evaluate the effect of rosuvastatin on each ion channel because the aim of our non-clinical study was to confirm the results from real-world data. Therefore, we only measured cardiac action potentials, which are widely used in vitro assays to assess QT risk. The effect of rosuvastatin on each ion channel and mechanism study will be performed in a follow-up study. We also did not consider the exact dose of rosuvastatin of each patient as a covariate in clinical data analysis. Regarding the mechanisms underlying the pharmacokinetics and pharmacodynamics of the drug, the risk of drug-induced QT prolongation can be assessed by considering the exact drug dose. Finally, we could not analyze the mortality of those considered at elevated risk of QT prolongation. Owing to the characteristics of the claim data with low reliability and precision, the selection of patients who are at high risk of QT prolongation was limited. If this had been possible, we could have determined whether exposure to rosuvastatin affected the mortality rate in high-risk patients.

In conclusion, our observations indicate a potential risk of QT prolongation with rosuvastatin use in real-world settings. Experimental data suggested that rosuvastatin may influence the action potential of hiPSC-CMs under laboratory conditions. However, despite these findings, long-term use of rosuvastatin was not correlated with increased mortality. Therefore, while these results provide a basis for caution, further research is required before definitive conclusions can be made for real-world application.

## Supplementary Information

Supplementary Information.

## Data Availability

The original contributions presented in the study are included in the article/supplementary material; further inquiries can be directed to the corresponding authors.
