# Supplementary material for: Evaluation of rosuvastatin-induced QT prolongation risk using real-world data, in vitro cardiomyocyte studies, and mortality assessment
Source: Sci Rep. 2023 May 19;13:8108. doi: 10.1038/s41598-023-35146-z (PMC10199059; doi:10.1038/s41598-023-35146-z)
Supplement: Supplementary file 1 — Supplementary Information. [file 41598_2023_35146_MOESM1_ESM.docx]

Supporting information

**Figure S1.** Flow chart for extracting study subjects for the case-control study. 58,505 QT prolongation cases and 386,077 controls were extracted to assess the risk of rosuvastatin-induced QT prolongation compared to the risk of atorvastatin-induced QT prolongation

Figure S2. The flow chart of extracting study subjects with a database from a single institute to assess the risk of prolonging the QT interval of rosuvastatin.


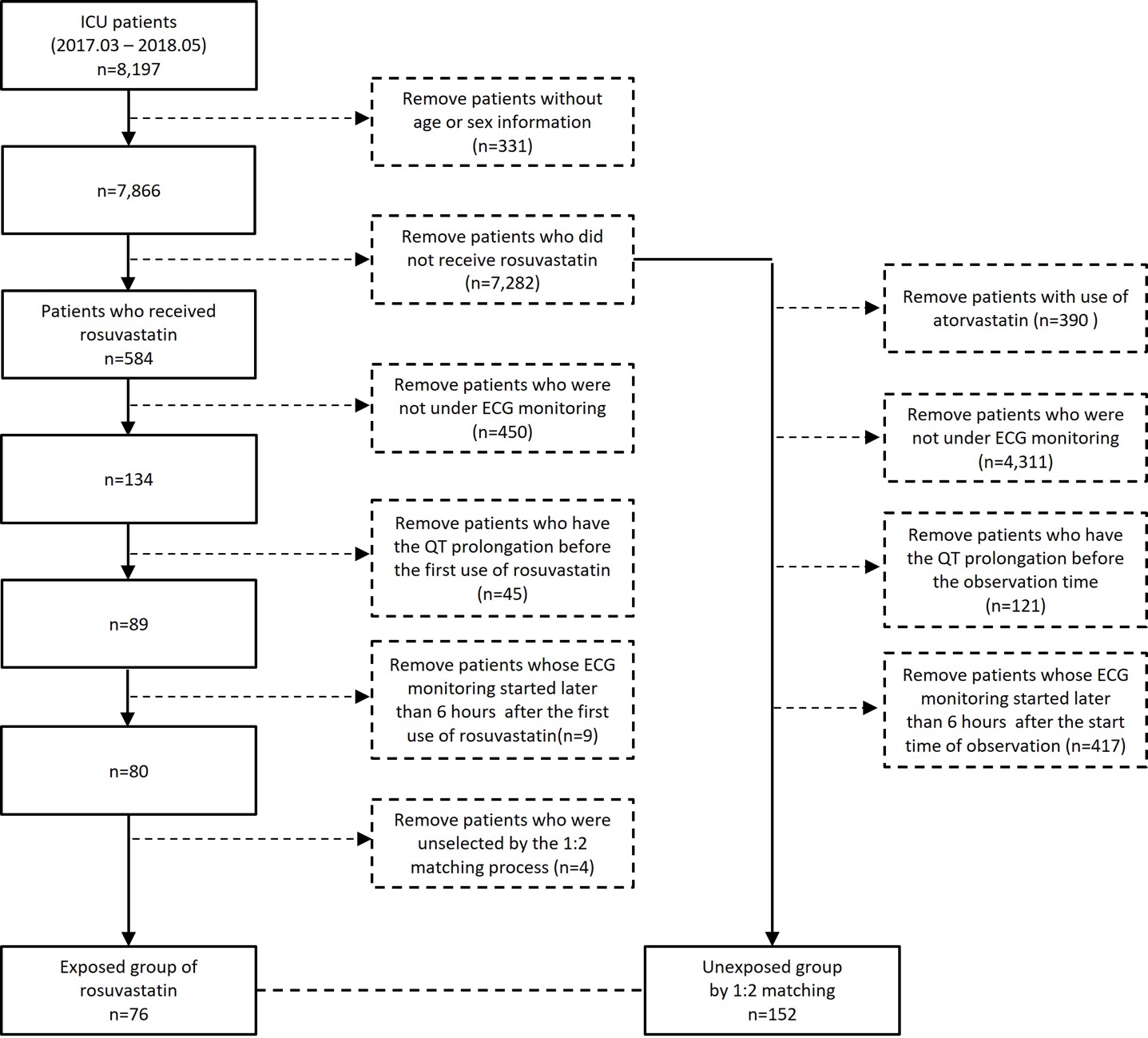


Figure S3. The flow chart of extracting study subjects with a database from a single institute to assess the risk of prolonging the QT interval of atorvastatin.


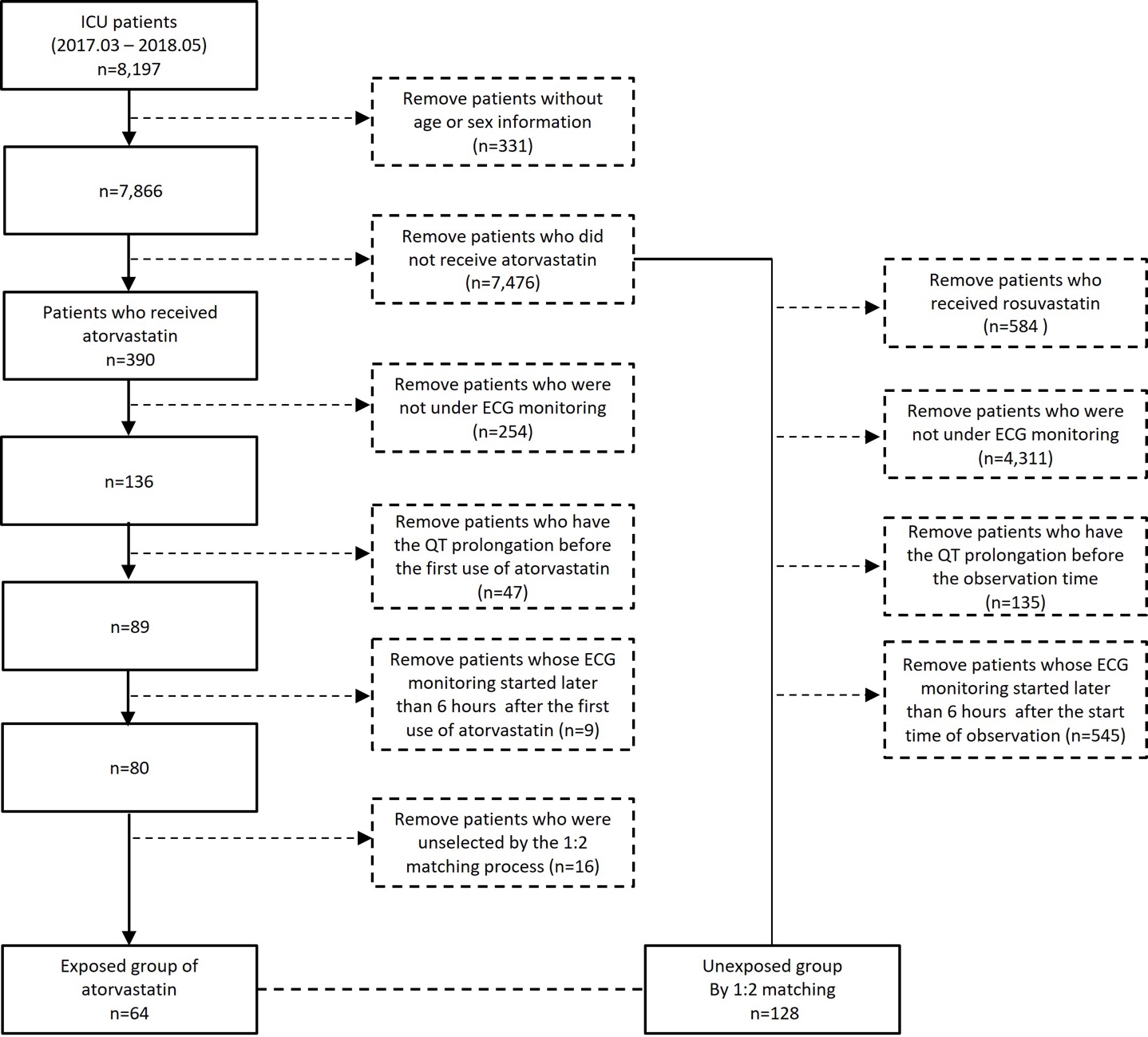


**Figure S4.** The flow chart of extracting study subjects for a survival study with the National Health Insurance Service (NHIS) database to assess the risk of QT prolongation induced by rosuvastatin compared to the risk of QT prolongation induced by atorvastatin


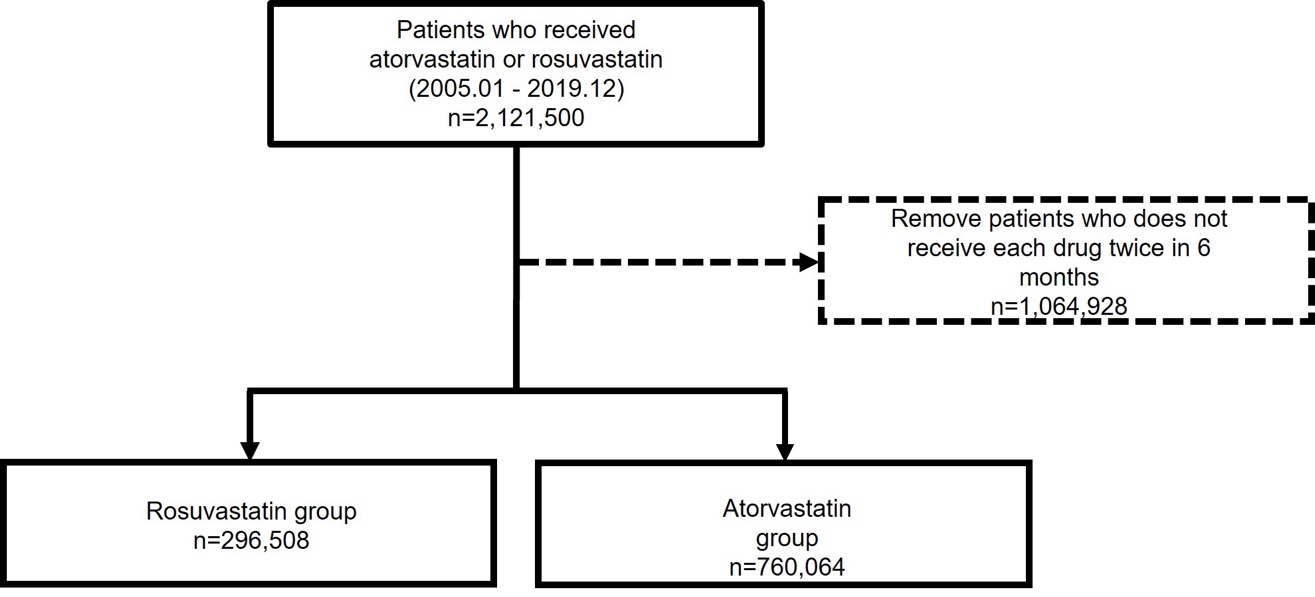


**Table S1.** The list of comorbidities and their ICD-10 cods known as risk factors of QT interval prolongation used in the analysis to correct bias

| Comorbidity | ICD-10 Code |
| --- | --- |
| Myocardial infarction | I21.x, I22.x, I25.2 |
| Congestive heart failure | I09.9, I11.0, I13.0, I13.2, I25.5, I42.0, I42.5–I42.9, I43.x, I50.x, P29.0 |
| Ischemic stroke | I63, G45 |
| Hemorrhagic stroke | I60, I61, I62 |
| Diabetes | E10.x E11.x E12.x E13.x |
| hypothyroidism | E00.x–E03.x, E89.0 |
| renal disease | I12.0, I13.1, N03.2–N03.7, N05.2– |
|  | N05.7, N18.x, N19.x, N25.0, Z49.0– |
|  | Z49.2, Z94.0, Z99.2 |
| Acquired Immune Deficiency Syndrome (AIDS) | B20.x–B22.x, B24.x |
| Alcohol abuse | F10, E52, G62.1, I42.6, |
|  | K29.2, K70.0, K70.3, |
|  | K70.9, T51.x, Z50.2, |
|  | Z71.4, Z72.1 |
| Drug abuse | F11.x–F16.x, F18.x, F19.x, Z71.5, Z72.2 |
| Liver disease | B18.x, K70.0–K70.3, K70.9, |
|  | K71.3–K71.5, K71.7, K73.x, K74.x, |
|  | K76.0, K76.2–K76.4, K76.8, K76.9, |
|  | Z94.4 |
| Severe liver disease | I85.0, I85.9, I86.4, I98.2, K70.4, |
|  | K71.1, K72.1, K72.9, K76.5, K76.6, |
|  | K76.7 |

**Table S2.** The list of concomitant drugs known to prolong QT interval used in the analysis

| Drug group | Drug names |
| --- | --- |
| Antiarrhythmic | quinidine, procainamide, disopyramide, flecainide, propafenone, amiodarone, dronedarone, vernakalant, sotalol, dofetilide, ibutilide |
| Antianginal | ranolazine, ivabradine |
| Anticholinergic | solifenacin, tolterodine |
| Antimallarials | artemether, artemether-lumefantrine, chloroquine, halofantrine, lumefantrine, delamanid, hydroxychloroquine, mefloquine, primaquine |
| Antituberculous | Bedaquiline |
| Antifungals | fluconazole, itraconazole, ketoconazole, posaconazole, voriconazole |
| Fluoroquinolone antibiotics | ciprofloxacin, gatifloxacin, levofloxacin, moxifloxacin, ofloxacin, sparfloxacin |
| HIV antiretrovirals | efavirenz, lopinavir-ritonavir, saquinavir, Atazanavir, nelfinavir, rilpivirine |
| Macrolide antibiotics | erythromycin, clarithromycin, roxithromycin, telithromycin |
| Antihistamines | astemizole, bilastine, hydroxyzine, terfenadine |
| Antineoplastic drugs | arsenic trioxide, bendamustine, capecitabine, ceritinib, cesium chloride, crizotinib, dasatinib, eribulin, fluorouracil, inotuzumab ozogamicin, nilotinib, lapatinib, lenvatinib, osimertinib, oxaliplatin, panobinostat, pazopanib, ribociclib, romidepsin, sorafenib, sunitinib, tegafur, toremifene, trifluridine-tipiracil, vandetanib, vemurafenib, vorinostat |
| Anesthetic/sedative | chloral hydrate, propofol |
| Opioids | buprenorphine, hydrocodone, loperamide, methadone |
| Bronchodilators (beta-agonists) | arformoterol, albuterol, formoterol, levalbuterol, indacaterol, olodaterol, salmeterol, terbutaline, vilanterol |
| Antidiarrheal | Loperamide |
| Antiemetics | ondansetron, granisetron, dolasetron, droperidol |
| Gastrointestinal Promotility | cisapride, domperidone, metoclopramide |
| GnRH¹ | buserelin, degarelix, goserelin, histrelin, leuprolide, triptorelin |
| Neurologic drugs | apomorphine, deutetrabenazine, donepezil, ezogabine, fingolimod, pimavanserin, tetrabenazine |
| Antipsychotics | chlorpromazine, haloperidol, levosulpiride, methotrimeprazine(levomepromazine), pimozide, sulpiride, thioridazine, amisulpride, aripiprazole, asenapine, clozapine, cyamemazine, flupentixol, iloperidone, melperone, olanzapine, paliperidone, perphenazine, pimavanserin, pipamperone, quetiapine, risperidone, sertindole, tiapride, ziprasidone |
| TCAs² | amitriptyline, amoxapine, clomipramine, desipramine, doxepin, imipramine, maprotiline, nortriptyline, protriptyline, trimipramine |
| SSRI³ | citalopram, escitalopram, fluoxetine, fluvoxamine, paroxetine, sertraline |
| Vasodilator drugs | bepridil, cilostazol |

*¹ Gonadotropin-releasing hormone agonists and antagonists*

*² Tricyclic and tetracyclic antidepressants*

*³ Selective serotonin reuptake inhibitors*

**Table S3.** The baseline characteristics of subjects of rosuvastatin analysis for the survival study

|  | | Exposed | Non-exposed | *p*-value |
| --- | --- | --- | --- | --- |
| Total | | 76 | 152 |  |
| QT prolongation (%) | | 14 (18.4) | 19 (12.5) | 0.318 |
| Sex |  | |  | 0.960 |
| Male, n (%) | | 51 (67.1) | 104 (68.4) |  |
| Female, n (%) | | 25 (32.9) | 48 (31.6) |  |
| Age, Mean (SD) | | 62.91 (12.36) | 63.03 (12.44) | 0.946 |
| Age, n (%) | |  |  | 1.000 |
| -29 | | 1 (1.3) | 2 (1.3) |  |
| 30-39 | | 12 (15.8) | 24 (15.8) |  |
| 40-49 | | 21 (27.6) | 42 (27.6) |  |
| 50-59 | | 15 (19.7) | 30 (19.7) |  |
| 60-69 | | 19 (25.0) | 36 (23.7) |  |
| 70- | | 8 (10.5) | 18 (11.8) |  |
| Serum potassium level (mEq/l), Mean (SD) | | 4.00 (0.43) | 3.91 (0.58) | 0.229 |
| Serum calcium level (mg/dl), Mean (SD) | | 8.72 (0.72) | 8.23 (0.88) | <0.001 |
| Comorbidity | |  |  |  |
| Myocardial infarction, n (%) | | 43 (56.6) | 2 (1.3) | <0.001 |
| Congestive heart failure, n (%) | | 12 (15.8) | 3 (2.0) | <0.001 |
| Ischemic stroke, n (%) | | 4 (5.3) | 6 (3.9) | 0.909 |
| Hemorrhagic stroke, n (%) | | 1 (1.3) | 7 (4.6) | 0.373 |
| Diabetes mellitus, n (%) | | 17 (22.4) | 8(5.3) | <0.001 |
| Renal disease, n (%) | | 7 (9.2) | 8 (5.3) | 0.395 |
| Alcohol abuse, n (%) | | 1 (1.3) | 1 (0.7) | 1.000 |
| Drug abuse, n (%) | | 0 (0.0) | 2 (1.3) | 0.802 |
| Liver disease, n (%) | | 0 (0.0) | 2 (1.3) | 0.802 |
| Severe liver disease, n (%) | | 0 (0.0) | 3 (2.0) | 0.538 |
| Concomitant Drugs with known risk of TdP | |  |  |  |
| Antiarrhythmic | | 2 (2.6) | 0 (0.0) | 0.209 |
| Antimalarial | | 2(2.6) | 0 (0.0) | 0.209 |
| Anticholinergic | | 0 (0.0) | 1(0.7) | 1.000 |
| Fluoroquinolone antibiotics | | 2 (2.6) | 3 (2.0) | 1.000 |
| Macrolide antibiotics | | 3 (3.9) | 3 (2.0) | 0.661 |
| Anesthetic/sedative | | 1 (1.3) | 10 (6.6) | 0.155 |
| Gastrointestinal Promotility | | 6 (7.9) | 5 (3.3) | 0.229 |
| Antipsychotics | | 3 (3.9) | 8 (5.3) | 0.913 |
| TCA | | 1 (1.3) | 0 (0.0) | 0.723 |
| Vasodilator drugs | | 2 (2.6) | 2 (1.3) | 0.858 |

**Table S4.** The baseline characteristics of subjects of atorvastatin analysis for the survival study

|  | Exposed | Non-exposed | *p*-value |
| --- | --- | --- | --- |
| Total | 64 | 128 |  |
| QT prolongation (%) | 10 (15.6) | 18 (14.1) | 0.942 |
| Sex |  |  | 1 |
| Male, n (%) | 40 (62.5) | 80 (62.5) |  |
| Female, n (%) | 24(37.5) | 48 (37.5) |  |
| Age, Mean (SD) | 68.81 (14.02) | 68.77 (13.91) | 0.985 |
| Age, n (%) |  |  | 1 |
| -29 | 3 (4.7) | 6 (4.7) |  |
| 30-39 | 2 (3.1) | 4 (3.1) |  |
| 40-49 | 12 (18.8) | 24 (18.8) |  |
| 50-59 | 12 (18.8) | 23 (18.0) |  |
| 60-69 | 18 (28.1) | 38 (29.7) |  |
| 70- | 17 (26.6) | 33 (25.8) |  |
| Serum potassium level (mEq/l), Mean (SD) | 3.89 (0.51) | 4.00 (0.55) | 0.151 |
| Serum calcium level (mg/dl), Mean (SD) | 8.51 (0.77) | 8.32 (0.87) | 0.15 |
| Comorbidity |  |  |  |
| Myocardial infarction, n (%) | 20 (31.2) | 1 (0.8) | <0.001 |
| Congestive heart failure, n (%) | 2 (3.1) | 6 (4.7) | 0.898 |
| Ischemic stroke, n (%) | 10 (15.6) | 8 (6.2) | 0.066 |
| Hemorrhagic stroke, n (%) | 5 (7.8) | 4 (3.1) | 0.277 |
| Diabetes mellitus, n (%) | 7 (10.9) | 7 (5.5) | 0.28 |
| Renal disease, n (%) | 4 (6.2) | 6 (4.7) | 0.909 |
| Alcohol abuse, n (%) | 0 (0.0) | 1 (0.8) | 1 |
| Liver disease, n (%) | 0 (0.0) | 1 (0.8) | 1 |
| Severe liver disease, n (%) | 0 (0.0) | 2 (1.6) | 0.802 |
| Concomitant Drugs with known risk of TdP |  |  |  |
| Antiarrhythmic | 3 (4.7) | 2 (1.6) | 0.423 |
| Antifungals | 2 (3.1) | 0 (0.0) | 0.209 |
| Fluoroquinolone antibiotics | 1 (1.6) | 3 (2.3) | 1 |
| Macrolide antibiotics | 6 (9.4) | 5 (3.9) | 0.227 |
| Antihistamines | 1 (1.6) | 0 (0.0) | 0.723 |
| Anesthetic/sedative | 3 (4.7) | 11 (8.6) | 0.492 |
| Bronchodilators | 1 (1.6) | 0 (0.0) | 0.723 |
| Gastrointestinal Promotility | 2 (3.1) | 1 (0.8) | 0.537 |
| Neurologic drugs | 1 (1.6) | 0 (0.0) | 0.723 |
| Antipsychotics | 2 (3.1) | 10 (7.8) | 0.343 |
| TCA | 0 (0.0) | 1 (0.8) | 1 |
| SSRI | 1 (1.6) | 2 (1.6) | 1 |
| Vasodilator drugs | 1 (1.6) | 0 (0.0) | 0.723 |

**Table S5**. Baseline characteristics of patients included in the case-control study

|  |  | Case | Control | *p*-value |
| --- | --- | --- | --- | --- |
| Total | | 58,505 | 386,077 |  |
| Sex | |  |  | < 0.001 |
|  | Men, n (%) | 29,856 (51.0) | 189,146 (49.0) |  |
|  | Women, n (%) | 28,649 (49.0) | 196,931 (51.0) |  |
| Age, n (%) | |  |  | < 0.001 |
|  | ≤ 39 | 12,410 (21.2) | 170,803 (44.2) |  |
|  | 40–49 | 8,270 (14.1) | 69,547 (18.0) |  |
|  | 50–59 | 10,264 (17.5) | 59,443 (15.4) |  |
|  | 60–69 | 10,880 (18.6) | 47,180 (12.2) |  |
|  | 70–79 | 11,217 (19.2) | 30,009 (7.8) |  |
|  | 80–89 | 4,876 (8.3) | 8,451 (2.2) |  |
|  | ≥ 90 | 588 (1.0) | 644 (0.2) |  |
| Serum potassium level, n (%) | |  |  | < 0.001 |
|  | 0–3.4 mEq/l | 6,177 (10.6) | 9,216 (2.4) |  |
|  | 3.5–3.7 mEq/l | 8,030 (13.7) | 27,874 (7.2) |  |
|  | 3.8–4.0 mEq/l | 11,375 (19.4) | 65,511 (17.0) |  |
|  | 4.1–4.3 mEq/l | 9,753 (16.7) | 71,529 (18.5) |  |
|  | 4.4–4.6 mEq/l | 5,441 (9.3) | 39,475 (10.2) |  |
|  | 4.7–4.9 mEq/l | 2,488 (4.3) | 15,156 (3.9) |  |
|  | ≥ 5.0 mEq/l | 15,241 (26.1) | 157,316 (40.7) |  |
| Serum calcium level, n (%) | |  |  | < 0.001 |
|  | 0–8.4 mg/dl | 11,959 (20.4) | 22,677 (5.9) |  |
|  | 8.5–8.8 mg/dl | 8,668 (14.8) | 38,244 (9.9) |  |
|  | 8.9–9.2 mg/dl | 10,973 (18.8) | 71,519 (18.5) |  |
|  | 9.3–9.6 mg/dl | 8,796 (15.0) | 70,188 (18.2) |  |
|  | 9.7–10.0 mg/dl | 3,833 (6.6) | 33,187 (8.6) |  |
|  | 10.1–10.4 mg/dl | 13,844 (23.7) | 147,910 (38.3) |  |
|  | ≥ 10.5 mg/dl | 432 (0.7) | 2,352 (0.6) |  |
| Comorbidity | |  |  |  |
|  | Myocardial infarction | 1,723 (2.9) | 3,457 (0.9) | < 0.001 |
|  | Congestive heart failure | 1,812 (3.1) | 2,390 (0.6) | < 0.001 |
|  | Ischemic stroke | 2,519 (4.3) | 6,332 (1.6) | < 0.001 |
|  | Hemorrhagic stroke | 1,480 (2.5) | 2,207 (0.6) | < 0.001 |
|  | Diabetes mellitus | 3,284 (5.6) | 12,364 (3.2) | < 0.001 |
|  | Hypothyroidism | 255 (0.4) | 1,999 (0.5) | < 0.001 |
|  | Renal disease | 2,068 (3.5) | 3,074 (0.8) | < 0.001 |
|  | AIDS/HIV | 22 (0.0) | 123 (0.0) | 0.552 |
|  | Alcohol abuse | 921 (1.6) | 1,145 (0.3) | < 0.001 |
|  | Drug abuse | 199 (0.3) | 392 (0.1) | < 0.001 |
|  | Liver disease | 1,350 (2.3) | 1,717 (0.4) | < 0.001 |
|  | Severe liver disease | 424 (0.7) | 282 (0.1) | < 0.001 |
|  | Rosuvastatin | 1,598 (2.7) | 3,434 (0.9) | < 0.001 |
|  | Atorvastatin | 786 (1.3) | 2,355 (0.6) | < 0.001 |
| Drug use count by rank, mean (SD) | | |  |  |
|  | Rank 1 drug | 0.88 (1.42) | 0.35 (0.84) | < 0.001 |
|  | Rank 2 drug | 0.49 (0.76) | 0.20 (0.50) | < 0.001 |
|  | Rank 3 drug | 0.16 (0.43) | 0.07 (0.27) | < 0.001 |
|  | Rank 4 drug | 0.19 (0.46) | 0.09 (0.32) | < 0.001 |

**Table S6.** Baseline characteristics of subjects from National Health Insurance Service (NHIS) data

|  |  | Atorvastatin | Rosuvastatin | *p*-value |
| --- | --- | --- | --- | --- |
| Total | | 760,064 | 296,508 |  |
| Sex | |  |  | < 0.001 |
|  | Men, n (%) | 351,215 (46.2) | 145,468 (49.1) |  |
|  | Women, n (%) | 408,849 (53.8) | 151,040 (50.9) |  |
| Age, mean (SD) | | 60.4 (12.5) | 59.6 (12.3) | < 0.001 |
| QT risk drugs, mean (SD) | |  |  |  |
|  | Conditional | 8.4 (10.4) | 7.3 (9.1) | < 0.001 |
|  | Possible | 4.3 (6.0) | 4.3 (6.0) | 0.495 |
|  | Known | 4.7 (5.8) | 4.1 (5.1) | < 0.001 |
| Comorbidity, n (%) | |  |  |  |
|  | AIDS | 360 (0.0) | 129 (0.0) | 0.437 |
|  | Alcohol abuse | 26,999 (3.6) | 9,179 (3.1) | < 0.001 |
|  | Cardiac arrhythmias | 49,679 (6.5) | 19,528 (6.6) | 0.355 |
|  | Cerebrovascular disease | 123,358 (16.2) | 38,656 (13.0) | < 0.001 |
|  | Congestive heart failure | 52,333 (6.9) | 19,901 (6.7) | 0.002 |
|  | Diabetes | 271,938 (35.8) | 109,481 (36.9)) | < 0.001 |
|  | Drug abuse | 282 (0.0) | 101 (0.0) | 0.496 |
|  | Fluid and electrolyte disorder | 39,574 (5.2) | 16,123 (5.4) | < 0.001 |
|  | Hemorrhage | 7,525 (1.0) | 2,187 (0.7) | < 0.001 |
|  | Hypertension | 476,281 (62.7) | 174,972 (59.0) | < 0.001 |
|  | Hypothyroidism | 54,662 (7.2) | 25,203 (8.5) | < 0.001 |
|  | Liver disease | 296,823 (39.1) | 119,225 (40.2) | < 0.001 |
|  | Moderate to severe liver disease | 5,260 (0.7) | 1,199 (0.4) | < 0.001 |
|  | Myocardial infarction | 19,849 (2.6) | 9,368 (3.2) | < 0.001 |
|  | Renal disease | 19,588 (2.6) | 6,583 (2.2) | < 0.001 |
|  | Valvular disease | 6,852 (0.9) | 2,637 (0.9) | 0.560 |

**Table S7.** Results of Cox-regression analysis using National Health Insurance Service (NHIS) data

|  | HR | 95% CI | *p*-value |
| --- | --- | --- | --- |
| Rosuvastatin | 0.95 | 0.89–1.01 | > 0.05 |
| Sex (ref. Male) |  |  |  |
| Female | 0.60 | 0.57–0.63 | > 0.05 |
| QT drug counts | 1.01 | 1.01 | < 0.001 |
| Comorbidities |  |  |  |
| AIDS | 0.90 | 0.43–1.89 | > 0.05 |
| Alcohol abuse | 1.08 | 0.95–1.22 | > 0.05 |
| Cardiac arrhythmias | 1.51 | 1.41–1.61 | < 0.001 |
| Cerebrovascular disease | 1.42 | 1.3–1.55 | < 0.001 |
| Congestive heart failure | 2.02 | 1.9–2.15 | < 0.001 |
| Diabetes | 1.45 | 1.37–1.52 | < 0.001 |
| Drug abuse | 2.08 | 1.23–3.53 | < 0.01 |
| Fluid and electrolyte disorder | 1.84 | 1.71–1.97 | < 0.001 |
| Hemorrhage | 1.40 | 1.19–1.64 | < 0.001 |
| Hypertension | 1.67 | 1.55–1.8 | < 0.001 |
| Hypothyroidism | 0.86 | 0.78–0.94 | < 0.01 |
| Liver disease | 0.80 | 0.76–0.84 | < 0.001 |
| Moderate to severe liver disease | 1.16 | 0.92–1.46 | > 0.05 |
| Myocardial infarction | 2.02 | 1.86–2.2 | < 0.001 |
| Renal disease | 2.22 | 2.04–2.43 | < 0.001 |
| Valvular disease | 1.79 | 1.56–2.05 | < 0.001 |
